# Bacteriophage pharmacodynamics studied in an *in vitro* pharmacokinetic model of infection

**DOI:** 10.1093/jacamr/dlaf213

**Published:** 2025-11-28

**Authors:** M L G Attwood, Pippa Griffin, Patryk Smorowinski, Alan Noel, Melissa Haines, Andrew Millard, Karen Adler, Martha Clokie, Alasdair Peter Macgowan

**Affiliations:** Bristol Centre for Antimicrobial Research & Evaluation (BCARE), Severn Infection Sciences, Pathology Sciences Building, North Bristol NHS Trust, Southmead Hospital, Westbury-on-Trym, Bristol BS10 5NB, UK; Bristol Centre for Antimicrobial Research & Evaluation (BCARE), Severn Infection Sciences, Pathology Sciences Building, North Bristol NHS Trust, Southmead Hospital, Westbury-on-Trym, Bristol BS10 5NB, UK; Bristol Centre for Antimicrobial Research & Evaluation (BCARE), Severn Infection Sciences, Pathology Sciences Building, North Bristol NHS Trust, Southmead Hospital, Westbury-on-Trym, Bristol BS10 5NB, UK; Bristol Centre for Antimicrobial Research & Evaluation (BCARE), Severn Infection Sciences, Pathology Sciences Building, North Bristol NHS Trust, Southmead Hospital, Westbury-on-Trym, Bristol BS10 5NB, UK; Becky Mayer Centre for Phage Research, Department of Genetics and Genome Biology, University of Leicester, Leicester LE1 7RH, UK; Becky Mayer Centre for Phage Research, Department of Genetics and Genome Biology, University of Leicester, Leicester LE1 7RH, UK; Becky Mayer Centre for Phage Research, Department of Genetics and Genome Biology, University of Leicester, Leicester LE1 7RH, UK; Becky Mayer Centre for Phage Research, Department of Genetics and Genome Biology, University of Leicester, Leicester LE1 7RH, UK; Bristol Centre for Antimicrobial Research & Evaluation (BCARE), Severn Infection Sciences, Pathology Sciences Building, North Bristol NHS Trust, Southmead Hospital, Westbury-on-Trym, Bristol BS10 5NB, UK

## Abstract

**Background:**

Bacteriophage therapy offers an alternative way to counter the menace of increasing antimicrobial resistance. Despite its use in clinical practice for many decades the basic tools to study the translational pharmacodynamics of phages are not available and it is recognized that lack of understanding of phage pharmacokinetics/pharmacodynamics (PK/PD) is a severe limitation in individual patient use and clinical trial design.

**Methods:**

Traditional *in vitro* PK/PD evaluation tools were used to assess the antibacterial effect of single exposures of a bacteriophage cocktail against four strains of *Escherichia coli* with potentially different patterns of response to phage. Initially, time–kill curves (TKCs) were performed over 48 h and subsequently a dilutional *in vitro* model (IVM) was used to assess the antibacterial effects over 72 h.

**Results:**

In TKCs, the four *E. coli* strains showed different patterns of kill and regrowth when exposed to phage, with two strains showing a sustained drop in bacterial viable count and two showing initial kill and regrowth. Using the IVM similar bacterial PD patterns were observed, and phage titre increased inversely and consistently with *E. coli* kill.

**Conclusions:**

An *in vitro* dilutional model can be used to study the antibacterial effect of a phage cocktail on *E. coli* showing strain-to-strain variation in bacterial killing and bacteriophage titre. Such models can be used to provide more nuanced information on phage PK/PD and translationally useful information for dosing in humans.

## Introduction

Since the discovery of the first antibiotic classes there have been concerns over the longevity of antibiotic efficacy, mainly related to antibiotic misuse and potential antibiotic resistance.^1^ Many *in vitro* tests have been designed to assess the efficacy of antimicrobials such as disc diffusion, micro broth dilution, time–kill studies and synergy testing.^2^ Although these tests aim to prevent incorrect antibiotic prescribing, they fail to assess bacterial–drug interactions from a dynamic drug concentration perspective. Time–kill curves (TKCs) can be used for semi-dynamic evaluations and are often performed when evaluating novel small molecules, but are not routinely performed in a diagnostic microbiology laboratory. TKCs are used initially to understand the relationship of antibacterial effect in order to establish a pharmacodynamic index (PDI); however, the model itself is limited by nutrients, single dose exposure and the employment of fixed antimicrobial concentrations.^3^  *In vitro* modelling systems overcome these issues and have been employed to provide more extensive and robust evaluations, which have been progressively used for drug development.^4^  *In vitro* models, dilutional or hollow fibre, both provide replenishment of nutrients, a precise bacterial infectious dose, human pharmacokinetic simulations, more detailed bacterial population analysis and the ability to determine emergence of resistance. These data allow the accurate determination of the dominant PDI, and its magnitude, which has good correlation with *in vivo* studies and informs optimum dosage and duration of antibiotic therapy.^5^

Despite rigorous evaluations for novel antibiotics or combination antibiotic therapies, antibiotic resistance continues to be a serious concern globally.^6^ This can be attributed to many factors: overuse of antibiotics in an unregulated manner, antibiotics use based on price rather than suitability, poor or outdated prescribing patterns, increasing numbers of highly populated regions, substandard antibiotic manufacture, ease of travel, natural bacterial evolution, and bacterial genetic mutation due to incorrect pharmacokinetic/pharmacodynamic (PK/PD) exposures.^7–9^ National actions plans (NAPs) have been put in place to address these issues in many countries. The UK NAP (Confronting Antimicrobial Resistance 2024 to 2029) states that: we should avoid unintentional antibiotic use; if antibiotics are required, we should optimize exposure (using PK/PD principles, therapeutic drug monitoring, etc.); we should innovate (potential new therapies); and we should continue to educate and practise antimicrobial stewardship practices.^10^

Phage therapy involves viruses that specifically target bacteria. This is not a new solution, as it was largely replaced by antibiotic use after World War II,^11^ but this type of therapy remains effective and has the potential to reduce the burden of antimicrobial resistance. Personalized phage therapy has been employed with good success in locations such as the Eliava Institute in Tbilisi, Georgia.^12^ Pre-defined phage therapy is a finished product that contains multiple phage strains and has been successfully used in agriculture and farming^13^ but could be applied in both human and veterinary medicine. More recently a large observational study of human phage therapy has shown both efficacy and safety, especially for the combination of bacteriophages and antimicrobials,^14^ and a randomized controlled trial in urinary tract infection showed non-inferiority of phage therapy alone compared with standard care.^15^ Despite these clinical findings, the optimal way of dosing phage in humans remains unclear. Here we describe the development of the PK/PD *in vitro* dilutional model for phage simulations in order to inform future optimum phage/antimicrobial dosing.

## Materials and methods

### Bacteria and media

Four clinical *Escherichia coli* strains from the collection at Southmead Hospital were used, one fluoroquinolone-sensitive strain (C1.15), one fluoroquinolone-resistant strain (C1.24), one ESBL-producing strain (C1.52) and one NDM-producing strain (C1.68). The antibiograms of each strain are shown in Table S1 (available as Supplementary data at *JAC-AMR* Online). The medium used for phage minimum inhibitory concentration (PMIC) determination^16^ was Mueller–Hinton broth II (BD 212322). The medium used for phage propagation challenge strains was Mueller–Hinton agar (Oxoid PO0152A).

### Bacterial killing curves

Bacterial TKCs were performed in 10 mL volumes in 30mL universal vessels (supplier - Camlab, product code - 1127949) providing sufficient nutrients to perform simulations of up to and including 48 h. Viable counts were determined using a spiral plater (Don Whitley, Shipley, UK). Aliquots were plated onto Mueller–Hinton agar plates for determination of viable counts (cfu/mL). The minimum level of bacterial detection was 1.0 ×10^2^ cfu/mL. The sampled aliquots were taken hourly (and enumerated for cfu/mL) from timepoint 0 to 8 h and then at every 24 h increment until the end of the simulation. All experiments were performed in triplicate.^17^

### In vitro PK model

An *in vitro* one-compartment PK model (IVM, Electrolab, Tewkesbury, UK) was used to simulate the target phage concentration at the site of infection (rather than phage oral dose titre), specifically the mean free-phage concentrations associated with experimental doses of phage retrieved in poultry.^18^ The apparatus has been described many times before for antibiotic investigations.^19^ The configuration of the model was largely the same as described. The central culture chamber (40 mL) was inoculated to reach a bacterial concentration of 1.5 ×10^6^ cfu/mL. This is connected to a main reservoir via silicone tubing to ensure nutrient addition/mimic flow rates (24 mL/h) for phage administration. Flow rates are determined by the speed of a peristaltic pump. A second peristaltic pump was used, which is connected to a waste vessel via silicone tubing (at 24.1 mL/h) to ensure the volume of the central chamber remains the same. The temperature was maintained at 37°C and the broth constantly agitated. Aliquots were obtained from the central chamber at hourly timepoints 0 to 8 h and then every 24 h until the end of the simulation. To validate the modelling system and to establish any phage-binding we employed *E. coli* bacterial strain ATCC 25922 and administered phage *E. coli* cocktail via two methods, bolus and infusion. Phage targets set were 1.5 × 10^2^, 10^4^ and 10^6^ pfu/mL after dosing, and recovery was within typical modelling parameters. In the main experimental simulations administration of phage was by bolus injection against four clinical *E. coli* strains, and all experiments were performed in triplicate.

### Phages and E. coli host strains

Phages JK08, 113 and UP17 and host *E. coli* strains MH10, B31 and EA2 were used. JK08, 113 and UP17 were combined in a cocktail at a ratio of 1:1:1.

### Phage propagation and titration phage propagation and titration

Challenge bacterial strain colonies were inoculated in Mueller–Hinton agar (Oxoid PO0152A) and grown overnight at 37°C aerobically. To prepare liquid culture, challenge strain colonies were inoculated into LB broth (Oxoid L3147) and grown overnight at 37°C agitated at 100 rpm. Phage propagation was performed by combining phages JK08, 113 and UP17 to the appropriate challenge strain at 1.0 × 10^7^ pfu/mL. Bacteria/phage mix cultures were incubated at 37°C at 100 rpm for 6.0 ± 1 h. Each bacteria/phage mix was centrifuged for 15 min at 4200 **g**, and supernatant filtered using (0.2 μ pore size filters). Phage titre was determined by serial dilution (using SM buffer) and plated via plaque assay techniques. Phage titre was stored at 4–8°C until use and appropriate dilutions performed as required.

### Phage direct spot testing

Bacterial cultures of appropriate challenge strains were grown overnight and then diluted 1/100 in LB and grown for 2 h to an 0D_550_ of 0.2. Then 500 µL of the culture was added to 8 mL 0.5% (w/v) LB agar kept molten at 55°C and poured onto Mueller–Hinton agar plates. Plates (double agar overlay assay plates) were left to set prior to testing aliquots from the central chamber. TKC or IVM aliquots were serially diluted to determine pfu/mL (testing series). Once serial dilution had been performed, 10 µL of each testing series was spotted directly onto previously made double agar overlay plaque assays. The plates were incubated overnight at 37°C and phage plaques enumerated.

### Data analysis

The antibacterial effect of each strain was described by the area under the bacterial kill curve [AUBKC, (log_10_cfu/mL).h], which was calculated according to the trapezoidal rule, to compare antibacterial effects. The data were plotted using the software package Graph Pad Prism Version 9 (Graph Pad, San Diego, CA, USA).

## Results

### PMICs

The phage cocktail produced the following PMICs: *E. coli* C1.15 = 1.0 ×10^4^ pfu/mL; C1.24 = 1.0 × 10^6^ pfu/mL; C1.52 = 1.0 ×10^5^ pfu/mL; and C1.68 = 1.0 × 10^5^ pfu/mL. The ranking of activity against the strains was C1.15 (cocktail most active), C1.52 and C1.68, then C1.24 (cocktail least active)

### Time–kill curves

**Figure 1.**
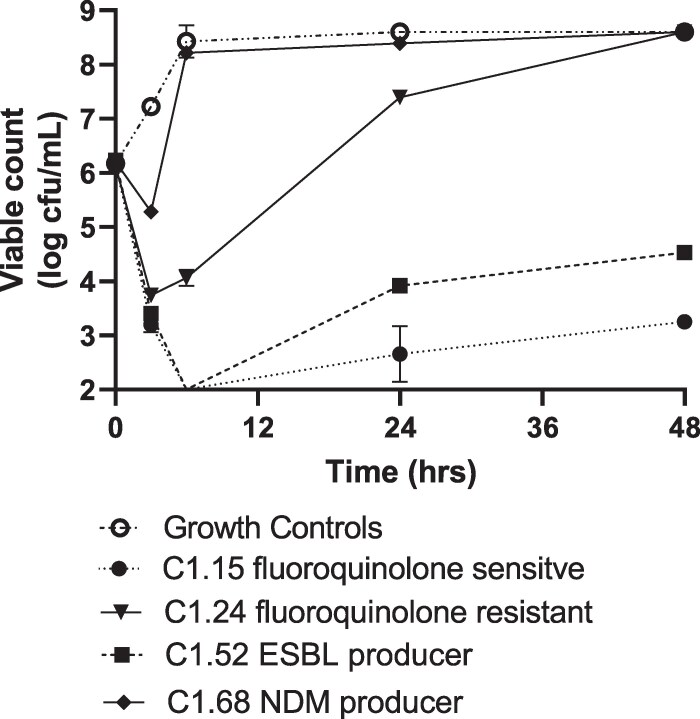
Antibacterial activity of the JK08,113, UP17 phage cocktail against four *E. coli* strains.

Figure 1 shows TKCs with a phage:bacteria ratio of 1:1 (bacterial cfu/mL target of 1.5 ×10^6^ and phage target of 1.5 × 10^6^ pfu/mL). Although the cocktail showed activity against all four strains, the pattern of bacterial kill over time was different. All strains showed initial killing with a 1–3-log drop in viable bacterial counts over the first 3 h; however, only two strains (C1.15 and C1.52) displayed further reduction in cfu/mL, with a 4-log drop at 6 h and suppression of bacterial counts below the initial inoculum until 48 h. Strains C1.24 and C1.68 showed regrowth from 3 h and bacterial counts were greater than the initial inoculum at 24 and 48 h. AUBKCs were calculated on transformed data and are shown in Table 1. The ranking of cocktail activity against the strains was C1.15 (cocktail most active), C1.52, C1.24 then C1.68 (cocktail least active)

**Table 1.** Antibacterial activity of the phage cocktail as measured by the area under the bacterial kill curve (AUBKC) against *E. coli*

Time of exposure, h	*E. coli* strain AUBKC, (log10 cfu/mL).h				
	Growth control	C1.15	C1.24	C1.52	C1.68
24	196.8 ± 2.8	63.68 ± 4.7	129.6 ± 1.7	75.77 ± 0.6	186.9 ± 1.1
48	403.3 ± 2.8	134.5 ± 7.9	321.5 ± 2.3	177.1 ± 1.2	390.8 ± 1.4

### In vitro model

**Figure 2.**
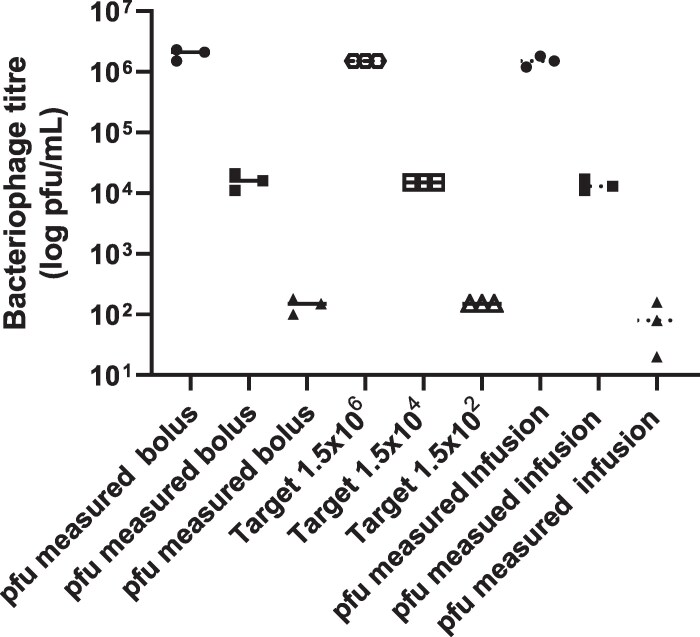
Assessment of phage binding to materials within the IVM system. Phage target titre versus measured phage titre recovery within 1 h for three bacteriophage titres.

**Figure 3.**
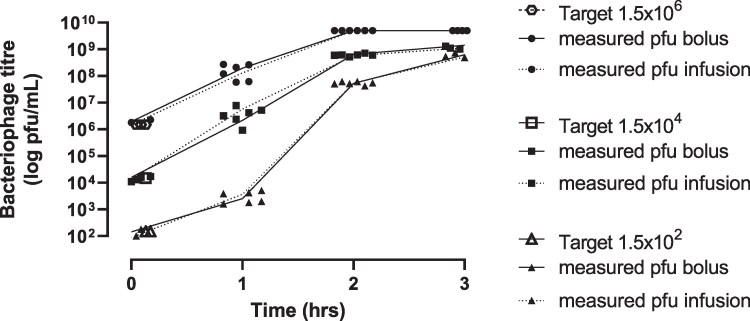
Assessment of phage binding and washout within the IVM system: phage dose and phage titre recovery over the first 3 h of the simulation.

Initial experiments showed the phage cocktail did not bind to the dilutional *in vitro* modelling system components when the cocktail was given as either bolus or infusion administration (Figure 2). In addition, washout was not a factor at the various target phage titres used; with 10^6^, 10^4^ and 10^2^ pfu/mL titres there was excellent recovery of phage (Figures 2 and 3).

**Figure 4.**
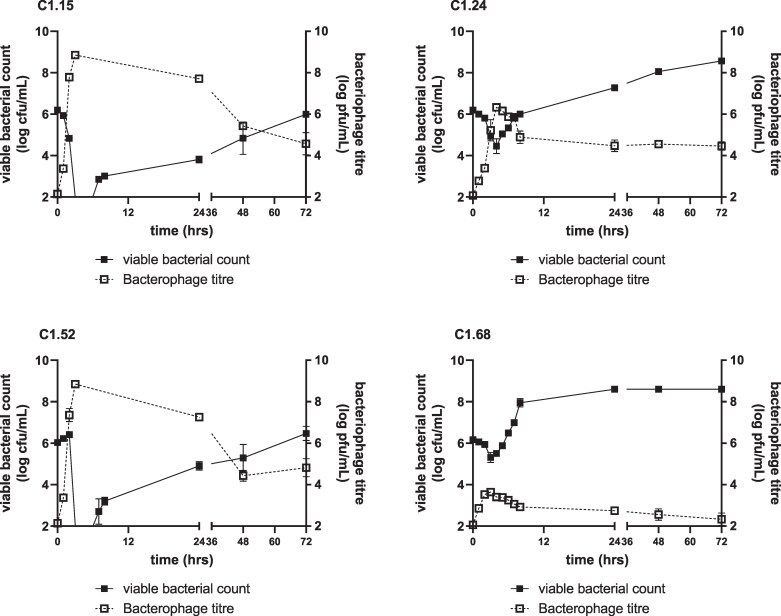
Antibacterial effect of a single exposure of bacteriophage cocktail on *E. coli* strains C1.15, C1.24, C1.52 and C1.68.

Figure 4 shows growth curves of the four *E. coli* strains exposed to a phage cocktail dose of 1.5 × 10^2^ pfu/mL, bacterial inoculum 1.5 × 10^6^ cfu/mL. Unsurprisingly the IVM model data mirror the TKC bacterial PD with one phage dose. C1.15 and C1.52 displayed >4-log reduction within the first 6 h of exposure, followed by bacterial regrowth to a bacterial load of approximately the initial inoculum by 72 h (Figure 2). With *E. coli* C1.15 and C1.52 phage titres increased significantly in the first 1–2 h, during which the inundation point (optimum phage titre to bacterial cfu ratio) was crossed (phage titre pfu exceeded bacterial cfu/mL) after 1–2 h exposure. C1.24 showed a gradual reduction in cfu/mL with a 1–2-log drop by 4 h; bacterial burden then increased until the end of the simulation. Inundation point (C1.24) was reached at 3 h, with phage titre increasing gradually until 4 h. C1.68 showed minimal bacterial reduction with phage titre, with a slight decrease in bacterial burden with an inverse relationship of phage titre for hours 2–3; however, phage titre remained low throughout the remainder of the simulation.

**Figure 5.**
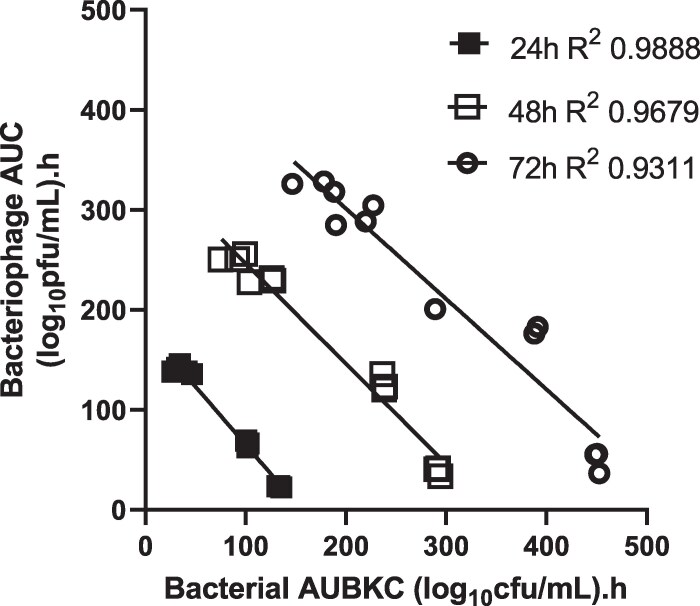
Relationship between antibacterial effect measured by the area under the bacterial kill curve (AUBKC) and bacteriophage exposure measured by the area under the bacteriophage titre time curve.

Figure 5 shows the relationship between the area under the bacteriophage concentration–time curve as a measure of total bacteriophage exposure and the AUBKC as a measure of bacterial kill. At all three timepoints analysed (24 h, 48 h or 72 h), as the phage exposure increased the bacterial kill increased. The ranking of cocktail activity against the strains in the IVM was C1.15 (cocktail most active; AUBKC at 24 h 32.8 ± 1.4), C1.52 (AUBKC at 24 h 44.8 ± 3.4), C1.24 (AUBKC at 24 h 101.9 ± 1.2) then C1.68 (AUBKC at 24 h 133.6 ± 2.0, cocktail least active).

## Discussion

In this study the *in vitro* PK/PD methodologies developed for small molecule antimicrobial evaluations have been shown to be effective tools to assess phage antibacterial effects, supporting previous work.^16^ The results from TKCs performed with single phage exposures were in close agreement with similar single dose exposures in our *in vitro* PK model. The PMIC methodology may be useful for determining basic phage efficacy, allowing the nuances of the phage–pathogen interaction to be initially assessed prior to performance of potentially more translatable experiments using TKCs and finally *in vitro* models. These approaches are essential in the design of phage dosing regimens for clinical trials as well as understanding phage–antibiotic interactions. We could also leverage these types of models to help inform phage–phage interactions, which are often hard to determine prior to clinical trials. Direct spot testing, efficiency of plating and planktonic killing assays are essential, informative and integral when designing multiple receptor targets for assessing phage cocktail candidates. This is especially true when trying to overcome the issue of emergence of resistance, but these experimental procedures can only provide a snapshot of interaction. The *in vitro* models that we have described have the potential to thoroughly assess phage–phage interactions, bacterial–phage cocktail dynamics and emergence of resistance over an extended period of time to determine optimum dose. Phage cocktails are designed to extend their spectrum of action, i.e. including phage that have different receptor targets. What is unknown, however, is whether in an active bacterial infection where optimum phage receptor targets are not present, do phage–phage interactions prove to be more dynamic. A recent narrative review by Nang *et al*.^20^ highlighted the key role of further phage PK/PD in future phage patient therapy.

There are concerns that phage could not be studied within existing *in vitro* systems, specifically hollow-fibre systems (HFIM). HFIM is a system of pumps, tubing and microfibres that allows for the *in vitro* assessment of anti-infective compounds, mimicking human conditions. These systems are used widely for PK/PD evaluations; but there are concerns about their use in phage evaluations due to the microfibre component damaging phage physiology, and also the cost associated with single-use cartridges. Fibres within the hollow fiber infection models (HFIM) could (i) damage the phage tail fibres preventing the phage from being able to infect their hosts or (ii) phage may become bound to the membranes within the central compartment of the system. We acknowledge that this could be overcome by selecting appropriate cartridges (where materials, pore size and permeability vary) and appropriate sampling could mitigate some of these issues. Interaction with the model components will often be phage specific and incur significant additional expense for each phage or phage cocktail evaluation. Dilutional *in vitro* models offer a solution to possible phage damage, phage binding (in that there are no fibres present) and expense (dilutional models are not single use). Washout of bacteria and phage is a potential source of concern with dilutional models but has been shown to be vastly overstated as a technical issue in antibacterial small molecule PK/PD studies.^21^ Our data show that with this phage cocktail, binding within the model system is not a problem and washout does not affect either bacterial clearance or phage titres. In addition, we have shown that exposure to phage can be measured and related to pathogen killing in an *in vitro* model, which paves the way for more analytical approaches to define phage dosing schedules.

Further work should now be performed, such as phage dose/exposure escalation studies, as well as on the potential timing and effect of second and subsequent phage exposures. This will enable the effect of phage dosing regimens on bacterial kill, regrowth and resistance to phage to be explored and allow for more translationally useful information to be acquired. We acknowledge that these models contain no elements of immune response, but we have a good track record (over 35 years) of progressing small molecules through the drug development pathway using these methods and we see no reason why we cannot apply these methodologies to phage evaluations also.

Dilutional *in vitro* models also allow performance of extended simulations over up to 14 days, which will help determine optimum phage:bacterial ratios, the emergence of resistance of phage (from a phenotypic and genetic perspective), the effect of different administration routes, the effectiveness of multiple phage doses, and the combination of phage with antibiotics, studied in conditions more akin to human therapy.

Although there is PK information available for specific phage cocktails, there is a paucity of phage PK data at target site concentrations.^22^ If these data become available, it will then be possible to mimic these using *in vitro* models to accurately simulate appropriate half-lives or to add additional phages as required to mimic phage proliferation. Although it could be argued that perhaps PK at target sites is not required, if all successful phage cocktails proliferate at predictable and consistent rates that are proportional and dependent on burst size. We can, however, use IVMs to answer such questions.

Finally, while acknowledging that there is a great deal of development work left to do, it seems that dilutional *in vitro* models will be an essential element in determining optimum phage dose, dosing frequency and duration for use in humans.

## Supplementary Material

dlaf213_Supplementary_Data

## Transparency declarations

M.L.G.A. and A.P.M. hold research grants/activities with Merck, Shionogi, InfectoPharm, GSK, Roche, BioVersys, VenatoRx Pharmaceuticals, iFAST, Technomede, Oxford Drug Design, JPIAMR and NIHR; A.P.M. provides consultancy advice to Shionogi, Roche, Bicycle Therapeutics and BioVersys. All other authors declare no competing interests; M.H. as an Academic Clinical Lecturer is funded by the NIHR. The view expressed in this publication are those of the author(s) and not necessarily those of the NIHR, NHS of the UK Department of Health and Social Care.

## Supplementary data

Table S1 is available as Supplementary data at *JAC-AMR* Online.
