# Supplementary material for: Bacteriophage pharmacodynamics studied in an in vitro pharmacokinetic model of infection
Source: JAC Antimicrob Resist. 2025 Nov 28;7(6):dlaf213. doi: 10.1093/jacamr/dlaf213 (PMC12662236; doi:10.1093/jacamr/dlaf213)
Supplement: dlaf213_Supplementary_Data [file dlaf213_supplementary_data.docx]

**Supplementary Material**

Table S1 Ecoli strain antimicrobial susceptibilities

|  | E coli strain | | | |
| --- | --- | --- | --- | --- |
|  | C1.15 | C1.24 | C1.52 | C1.68 |
| co-amoxiclav | R | R | R | R |
| ceftazidime | S | S | R | R |
| ciprofloxacin | S | R | S | R |
| ceftriaxone | S | S | R | R |
| cefuroxime | S | S | R | R |
| ertapenem | S | S | S | R |
| gentamicin | R | R | S | R |
| meropenem | S | S | S | R |
| co-trimoxazole | S | R | R | R |
| piperacillin/tazobactam | S | S | R | R |
| amoxicillin | R | R | R | R |
| amikacin | NT | S | S | R |
| aztreonam | NT | S | R | R |
| cefixime | NT | S | R | R |
| cefepime | NT | S | R | R |
| tigecycline | NT | S | S | S |
| tobramycin | NT | R | S | R |

NT: Not tested
